# Z-Ligustilide Combined with Cisplatin Reduces PLPP1-Mediated Phospholipid Synthesis to Impair Cisplatin Resistance in Lung Cancer

**DOI:** 10.3390/ijms242317046

**Published:** 2023-12-01

**Authors:** Pengyu Geng, Jinhui Zhao, Qi Li, Xiaolin Wang, Wangshu Qin, Ting Wang, Xianzhe Shi, Xinyu Liu, Jia Chen, Hongdeng Qiu, Guowang Xu

**Affiliations:** 1 CAS Key Laboratory of Separation Science for Analytical Chemistry, Dalian Institute of Chemical Physics, Chinese Academy of Sciences, Dalian 116023, China; gengpy470@dicp.ac.cn (P.G.); zhaojh0714@dicp.ac.cn (J.Z.); liqi1808@dicp.ac.cn (Q.L.); wangxiaolin@dicp.ac.cn (X.W.); qinwangshu@dicp.ac.cn (W.Q.); wangting1830@dicp.ac.cn (T.W.); shixianzhe@dicp.ac.cn (X.S.); liuxy2012@dicp.ac.cn (X.L.); 2 Liaoning Province Key Laboratory of Metabolomics, Dalian 116023, China; 3 CAS Key Laboratory of Chemistry of Northwestern Plant Resources, Key Laboratory for Natural Medicine of Gansu Province, Lanzhou Institute of Chemical Physics, Chinese Academy of Sciences, Lanzhou 730000, China; jiachen@licp.cas.cn (J.C.); hdqiu@licp.cas.cn (H.Q.)

**Keywords:** cisplatin resistance, Z-Ligustilide, cell cycle arrest, cell apoptosis, phospholipid synthesis, PLPP1

## Abstract

Lung cancer is a malignant tumor with one of the highest morbidity and mortality rates in the world. Approximately 80–85% of lung cancer is diagnosed as non-small lung cancer (NSCLC), and its 5-year survival rate is only 21%. Cisplatin is a commonly used chemotherapy drug for the treatment of NSCLC. Its efficacy is often limited by the development of drug resistance after long-term treatment. Therefore, determining how to overcome cisplatin resistance, enhancing the sensitivity of cancer cells to cisplatin, and developing new therapeutic strategies are urgent clinical problems. Z-ligustilide is the main active ingredient of the Chinese medicine Angelica sinensis, and has anti-tumor activity. In the present study, we investigated the effect of the combination of Z-ligustilide and cisplatin (Z-ligustilide+cisplatin) on the resistance of cisplatin-resistant lung cancer cells and its mechanism of action. We found that Z-ligustilide+cisplatin decreased the cell viability, induced cell cycle arrest, and promoted the cell apoptosis of cisplatin-resistant lung cancer cells. Metabolomics combined with transcriptomics revealed that Z-ligustilide+cisplatin inhibited phospholipid synthesis by upregulating the expression of phospholipid phosphatase 1 (PLPP1). A further study showed that PLPP1 expression was positively correlated with good prognosis, whereas the knockdown of PLPP1 abolished the effects of Z-ligustilide+cisplatin on cell cycle and apoptosis. Specifically, Z-ligustilide+cisplatin inhibited the activation of protein kinase B (AKT) by reducing the levels of phosphatidylinositol 3,4,5-trisphosphate (PIP_3_). Z-ligustilide+cisplatin induced cell cycle arrest and promoted the cell apoptosis of cisplatin-resistant lung cancer cells by inhibiting PLPP1-mediated phospholipid synthesis. Our findings demonstrate that the combination of Z-Ligustilide and cisplatin is a promising approach to the chemotherapy of malignant tumors that are resistant to cisplatin.

## 1. Introduction

Lung cancer is the leading cause of cancer-related death worldwide. Non-small-cell lung cancer (NSCLC) is the main type of lung cancer, accounting for 80–85% of total lung cancer cases [1]. Due to the lack of obvious clinical symptoms and reliable biological markers in the early stage of lung cancer, most lung cancer patients are already in the advanced stage when they are diagnosed [2]. Currently, platinum-based chemotherapeutics are the main chemotherapeutic methods for the treatment of lung cancer, among which cisplatin is a common chemotherapy drug [3,4]. During treatment with cisplatin, drug resistance often occurs, resulting in chemotherapy having a poor effect and tumor recurrence [5,6]. A large number of studies have shown that multiple mechanisms are involved in cisplatin resistance, including the decreased intracellular accumulation of cisplatin and increased cisplatin efflux [7], enhanced DNA repair abilities [8], enhanced autophagy [9], inhibited apoptosis [10], inhibited ferroptosis [11], and abnormal lipid metabolism [12]. Therefore, exploring a drug that can effectively enhance the sensitivity of tumor cells to cisplatin and reduce their drug resistance will be a major breakthrough for the treatment of lung cancer.

Z-ligustilide belongs to the phthalide class, and mostly exists in the Angelica sinensis and Chuangxiong. It has anti-inflammatory, antioxidant and neuroprotective effects [13]. In recent years, plenty of evidence has shown that Z-ligustilide has anti-tumor activity in various tumors. For example, Yin et al. proved that Z-ligustilide reduces cell migration by decreasing the expressions of the Rho family of small GTPases (Rho GTPases) in glioblastoma [14]. Z-ligustilide induces cell death and oxidative stress via the epigenetic transcriptional regulation of nuclear factor-erythroid 2-related factor2 (Nrf2) in prostate cancer [15]. In regulating cell apoptosis, Yin et al. revealed that Z-ligustilide induces cell apoptosis by upregulating the expression of pro-apoptosis BCL-2 protein (BAX) and downregulating the expression of nuclear factor -kappa B (NFκB1) in bladder cancer [16]. However, Hsu et al. demonstrated that Z-ligustilide promotes apoptosis by activating endoplasmic reticulum (ER)-stress signaling in hypoxic oral cancer [17]. In lung cancer, Z-ligustilide has been demonstrated to inhibit cell proliferation and promote cell apoptosis by inhibiting glycolysis [18]. However, the role of Z-ligustilide in cisplatin-resistant lung cancer has not been reported yet, and the molecular mechanism remains unclear.

Metabolic reprogramming is a hallmark of malignancy [19]. Lipid metabolism reprogramming has gradually been recognized as a key mechanism for promoting cancer cell survival and proliferation [20,21], and is closely related to the development of drug resistance [22]. For instance, gefitinib-resistant and gefitinib-sensitive NSCLC cells exhibit distinct phospholipid compositions, revealing phospholipid remodeling during drug resistance [23]. Phosphatidylinositol 3, 4, 5-trisphosphate (PIP_3_) is a type of membrane phospholipid that functions as a second messenger. It can recruit 3-phosphoinositide-dependent protein kinase 1 (PDK1) and protein kinase B (AKT) to the plasma membrane, in which PDK1 phosphorylates threonine at position 308 of AKT protein, resulting in the activation of AKT [24]. Activated AKT can promote cell proliferation, cell survival, angiogenesis, glucose metabolism, lipid metabolism and inhibit apoptosis through phosphorating downstream effector proteins [25]. Moreover, the activation of AKT can increase drug resistance in various types of cancer [26]. Yu et al. found that baicalein reversed the cisplatin resistance of lung cancer by inhibiting apoptosis through the AKT/NFκB pathway [27]. Zhou et al. reported that glioma-associated oncogene 1 (GLI1) enhanced the drug resistance of acute myeloid leukemia by regulating the cell cycle via the AKT/serine/threonine kinase glycogen synthesis kinase (GSK)/cyclin-dependent kinase (CDK) pathway [28].

In this work, we first investigate the sensitivity and biological function of Z-ligustilide in cisplatin-resistant lung cancer cells, and further explore the related action mechanism of Z-ligustilide from the perspective of metabolic reprogramming. We confirm that Z-ligustilide combined with cisplatin (Z-ligustilide+cisplatin) reverses the resistance of cisplatin-resistant lung cancer cells by inducing cell cycle arrest and apoptosis. Specifically, we reveal that Z-ligustilide+cisplatin reduces the PIP_3_ level to inactivate AKT by inhibiting phospholipid synthesis.

## 2. Results

### 2.1. Z-Ligustilide Alleviated the Cisplatin Resistance of Cisplatin-Resistant Lung Cancer Cells

**Figure 1:**
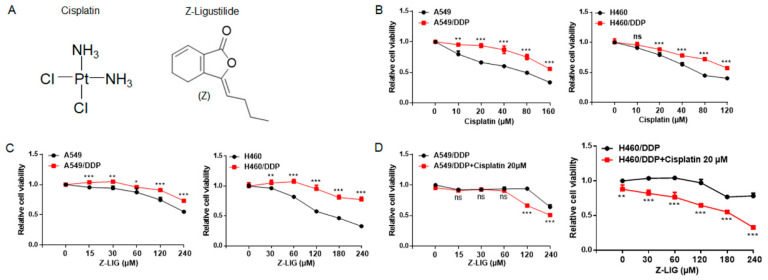
Z-ligustilide inhibited cell viability of cisplatin-resistant lung cancer cells. (**A**) Chemical structures of Cisplatin and Z-ligustilide (Z-LIG). (**B**) Cell viability of A549 cells and cisplatin-resistant A549 (A549/DDP) cells, H460 cells and cisplatin-resistant H460 (H460/DDP) cells after treatment with cisplatin. (**C**) Cell viability of A549 cells and A549/DDP, H460 cells and H460/DDP cells after treatment with Z-ligustilide. (**D**) Cell viability of A549/DDP cells and H460/DDP cells after treatment with Z-ligustilide+cisplatin. ns, not significant, * *p* < 0.05, ** *p* < 0.01 and *** *p* < 0.001.

The chemical structures of cisplatin and Z-ligustilide are shown in Figure 1A. The effects of cisplatin and Z-ligustilide on the cell viability of lung cancer cells and cisplatin-resistant lung cancer cells were investigated. The cell counting kit-8 (CCK-8) assay revealed that A549 and H460 cells were more sensitive to cisplatin than cisplatin-resistant A549 (A549/DDP) and cisplatin-resistant H460 (H460/DDP) cells (Figure 1B).

Moreover, Z-ligustilide was able to reduce the cell viability of A549 and H460 cells more than that of A549/DDP and H460/DDP cells (Figure 1C). Further, A549/DDP and H460/DDP cells with various concentrations of Z-ligustilide alone or in combination with cisplatin for 24 h were treated, and the CCK-8 assay demonstrated that 120 μM of Z-ligustilide alone reduced the cell viability of cisplatin-resistant lung cancer cells by less than 10%. However, Z-ligustilide combined with 20 μM of cisplatin could decrease the cell viability by more than 30% (Figure 1D). These results indicated that Z-ligustilide+cisplatin significantly decreased the cell viability of cisplatin-resistant lung cancer cells.

### 2.2. Z-Ligustilide+Cisplatin Induced Cell Cycle Arrest and Promoted Cell Apoptosis of Cisplatin-Resistant Lung Cancer Cells

**Figure 2:**
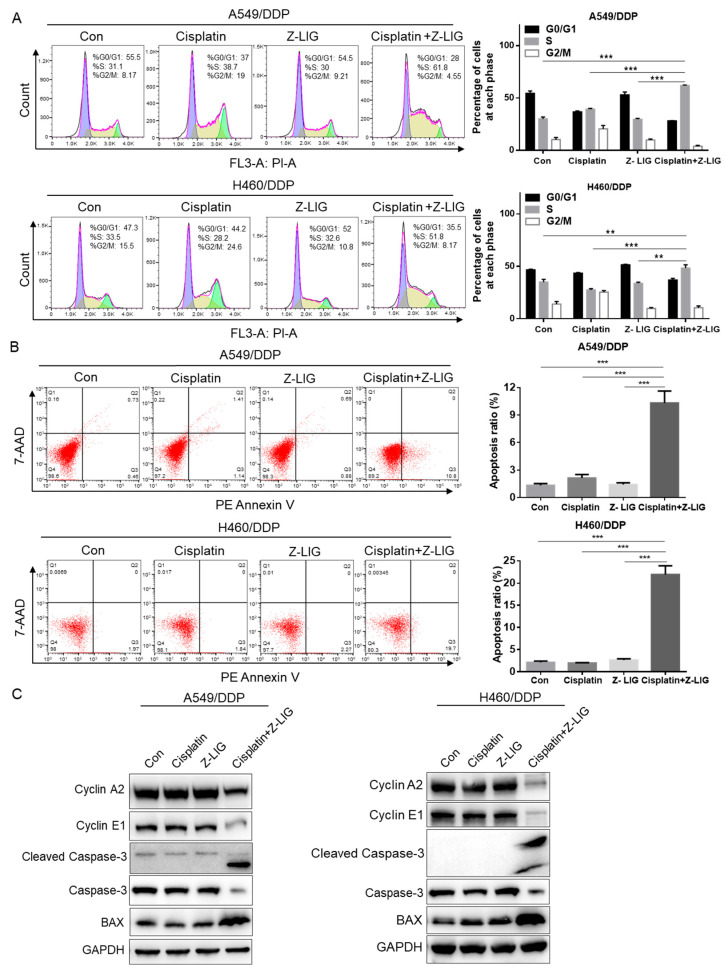
Z-ligustilide+cisplatin induced S phase arrest and promoted cell apoptosis. (**A**) Cell cycle analysis of A549/DDP and H460/DDP, A549/DDP and H460/DDP treated with cisplatin and Z-ligustilide alone or in combination for 24 h. (**B**) PE Annexin V apoptosis analysis of A549/DDP and H460/DDP, A549/DDP and H460/DDP treated with cisplatin and Z-ligustilide alone or in combination for 24 h. (**C**) Protein expression of Cyclin A2, Cyclin E1, Cleaved Caspase-3, Caspase-3 and BAX in A549/DDP and H460/DDP, A549/DDP and H460/DDP treated with cisplatin and Z-ligustilide alone or in combination. ** *p* < 0.01 and *** *p* < 0.001.

The effect of cisplatin and Z-ligustilide on cell proliferation was analyzed using a cell cycle assay experiment. The results indicated that Z-ligustilide+cisplatin induced cell cycle S-phase arrest in cisplatin-resistant lung cancer cells (Figure 2A). Considering that many drugs can induce cell cycle arrest at different stages, eventually leading to cell apoptosis [29,30], we examined the effect of Z-ligustilide and cisplatin on the apoptosis of cisplatin-resistant lung cancer cells. The control (Con), cisplatin, Z-ligustilide, and Z-ligustilide+cisplatin were used to treat the A549/DDP and H460/DDP cells, respectively. Cell apoptosis analysis confirmed that Z-ligustilide+cisplatin significantly increased the proportion of apoptotic cells compared to the two compounds alone (Figure 2B). Real-time PCR showed a sharp downregulation of the cell cycle regulatory proteins Cyclin A and Cyclin E, a significant upregulation of the apoptosis-related protein BAX, and the downregulation of Bcl-2 in A549/DDP and H460/DDP cells treated with Z-ligustilide+cisplatin (Figure S1). Western blot showed that the expressions of Cyclin A, Cyclin E and Caspase-3 were downregulated, and that the expressions of Cleaved Caspase-3 and BAX were upregulated in A549/DDP and H460/DDP cells treated with Z-ligustilide+cisplatin (Figure 2C). Taken together, these data indicate that Z-ligustilide+cisplatin plays an important role in inducing cell cycle arrest and cell apoptosis in cisplatin-resistant lung cancer cells.

### 2.3. Z-Ligustilide+Cisplatin Reduced Phospholipid Synthesis and Increased the Expression of PLPP1

**Figure 3:**
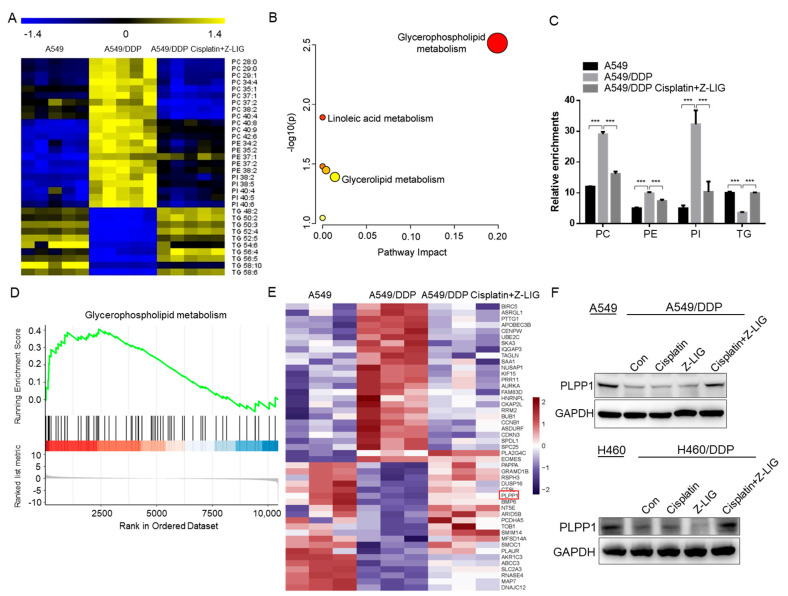
Z-ligustilide+cisplatin reduced the phospholipid contents and upregulated PLPP1 expression. (**A**) Heat map of the differential lipids in A549, A549/DDP cells and A549/DDP cells for Z-ligustilide+cisplatin. (**B**) Pathway enrichment analysis. (**C**) Relative enrichment of each lipid class. *** *p* < 0.001. (**D**) GSEA analysis of the RNA-seq data. The glycerophospholipid metabolism pathway–targeted genes show significant overlap. (**E**) Heat map for RNA–Seq analysis of significantly differentially expressed genes in A549 cells vs. A549/DDP cells vs. A549/DDP cells treated with Z-ligustilide+cisplatin. Red box represented the target gene. (**F**) Protein level of PLPP1 determined via Western blot.

As lipid metabolic disturbance is a crucial factor for promoting cancer cell survival and proliferation, we wondered whether the resistance of cisplatin-resistant lung cancer cells was associated with lipid metabolism reprogramming, and whether Z-ligustilide+cisplatin could regulate lipid metabolism. To this end, the lipid metabolic profile of A549 cells, A549/DDP cells and A549/DDP cells treated with the Z-ligustilide and cisplatin were analyzed using liquid chromatography-mass spectrometry (LC-MS). A total of 32 lipids exhibited significant changes among the three groups. A heat map of the significantly changed lipids showed that phospholipids obviously increased and triacylglycerols (TGs) decreased in A549/DDP cells compared with A549; however, A549/DDP cells treated with Z-ligustilide+cisplatin reversed the content of phospholipids and TGs (Figure 3A). Pathway enrichment analysis showed that glycerophospholipid metabolism was significantly altered (Figure 3B). Further analysis revealed that A549/DDP cells had higher levels of phosphatidylcholine (PC), phosphatidylethanolamine (PE) and phosphatidylinositol (PI), and lower levels of TG than A549 cells. However, Z-ligustilide+cisplatin greatly altered the content of PC, PE, PI and TG in A549/DDP cells (Figure 3C).

To investigate the key factors regulated by Z-ligustilide+cisplatin, we carried out RNA sequencing (RNA-seq) analysis of A549 cells, A549/DDP cells and A549/DDP cells treated with Z-ligustilide+cisplatin. Gene set enrichment analysis (GSEA) of RNA-seq data revealed that the genes involved in glycerophospholipid metabolism were highly enriched (Figure 3D). Through RNA sequencing analysis, phospholipid phosphatase 1 (PLPP1) experienced a significant change in A549 cells, A549/DDP cells and A549/DDP cells treated with Z-ligustilide+cisplatin (Figure 3E). The mRNA level and protein expression of PLPP1 were downregulated in A549/DDP and H460/DDP cells compared with those in A549 and H460 cells, but Z-ligustilide+cisplatin reversed the expression of PLPP 1 (Figure S2 and Figure 3F). 

**Figure 4:**
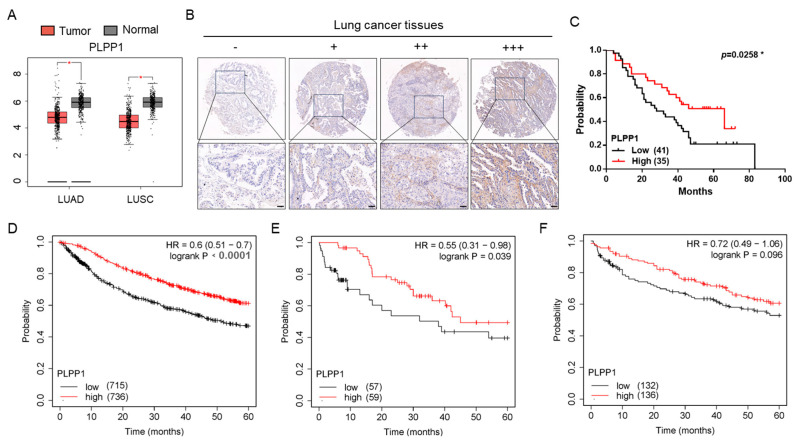
PLPP1 expression was low and associated with poor prognosis in lung cancer. (**A**) PLPP1 expression was detected in LUAD, LUSC, and corresponding normal tissues. LUAD (num (T) = 483; num (N) = 347). LUSC (num (T) = 486; num (N) = 338). * *p* < 0.05. (**B**) Representative images of the immunohistochemical staining of PLPP1 in lung cancer samples. Scale bars, 50 μm. −: negative expression; +: lower expression; ++: moderate expression; +++: high expression. The score criteria are described in detail in Materials and Methods. (**C**) The association between the PLPP1 protein expression and overall survival in patients with lung cancer. Low PLPP1 expression (n = 41), high PLPP1 expression (n = 35). * *p* < 0.05. (**D**) The correlation between PLPP1 expression and overall survival in 1451 lung cancer patients. (**E**) The correlation between PLPP1 expression and overall survival in 116 lung cancer patients administered chemotherapy. (**F**) The correlation between PLPP1 expression and overall survival in 268 lung cancer patients without chemotherapy.

Next, we explored the expression of PLPP1 in clinical samples. The Cancer Genome Atlas (TCGA) and Genotype-Tissue Expression (GTEx) data set analysis showed that PLPP1 had a lower expression in 483 lung adenocarcinoma (LUAD) and 486 lung squamous cell carcinoma (LUSC) than in corresponding normal tissues (Figure 4A). The immunohistochemical staining of PLPP1 in 76 lung cancer tissue samples was further performed. The results revealed that 75% (57/76) of lung cancer samples showed positive PLPP1staining. Among these positive samples, 23 specimens showed lower expression, 32 moderate expression, and only 2 high expression. The typical PLPP1 images are shown in Figure 4B. Lung cancer patients with a low protein expression of PLPP1 had poor overall survival (Figure 4C). Furthermore, we used the database of the Kaplan–Meier (KM) plotter to analyze the association between PLPP1 mRNA expression and the prognosis of lung cancer. We found that lung cancer patients with low PLPP1 expression had a shorter overall survival than patients with high PLPP1 expression during the 60-month follow-up (Figure 4D). A more detailed analysis revealed that the overall survival of lung cancer patients with low PLPP1expression was shorter than patients with high PLPP1 expression in the chemotherapy group (Figure 4E). However, overall survival was not significantly different in the non-chemotherapy group (Figure 4F). Collectively, these results indicated that PLPP1 expression was low and associated with poor prognosis in lung cancer.

### 2.4. Z-Ligustilide+Cisplatin Reversed Cisplatin Resistance through PLPP1-Mediated Phospholipid Synthesis

**Figure 5:**
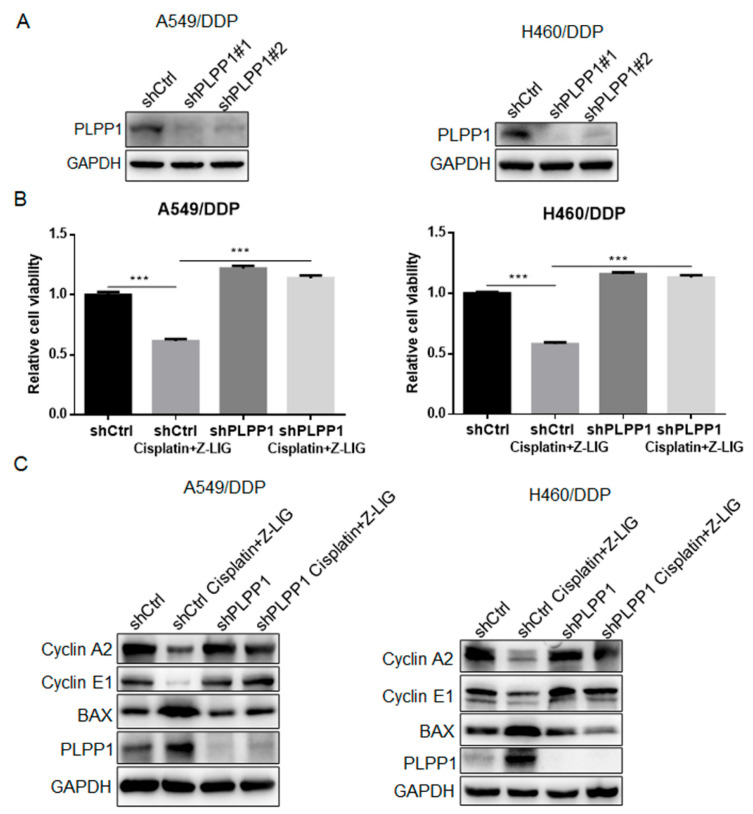
Z-ligustilide+cisplatin induced the reversal of resistance mediated by PLPP1. (**A**) Protein expression of PLPP1 in shCtrl and PLPP1 knockdown A549/DDP and H460/DDP cells, respectively. (**B**) Cell viability was measured in shCtrl and PLPP1 knockdown A549/DDP and H460/DDP cells with or without the Z-ligustilide and cisplatin combination treatment, *** *p* < 0.001. (**C**) Relative protein expressions in shCtrl and PLPP1 knockdown A549/DDP and H460/DDP cells with or without the Z-ligustilide and cisplatin combination treatment.

To investigate whether PLPP1 participated in the effect of Z-ligustilide+cisplatin on the resistance of cisplatin-resistant lung cancer cells, we knocked down the expression of PLPP1 in A549/DDP and H460/DDP cells, and the mRNA level and protein expression of PLPP1 were examined using real-time PCR and Western blot analysis (Figure S3 and Figure 5A). PLPP1 silence restored the Z-ligustilide+cisplatin-induced effects on cell viability, cell cycle and cell apoptosis in A549/DDP and H460/DDP cells (Figure 5B,C). The data demonstrated that PLPP1 was necessary for cell cycle arrest and cell apoptosis induced by Z-ligustilide+cisplatin.

**Figure 6:**
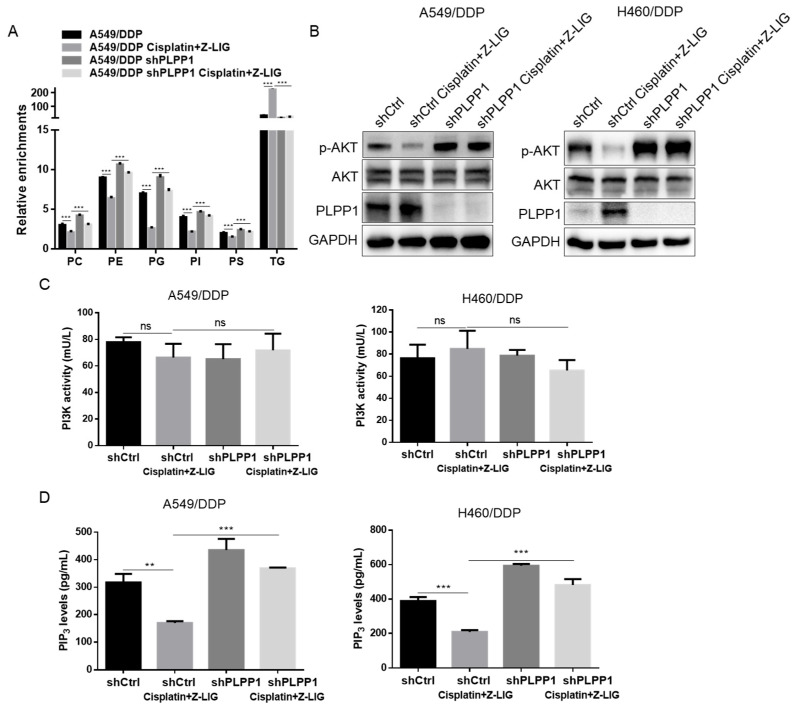
Z-ligustilide+cisplatin induced the inactivation of AKT by inhibiting PLPP1-mediated phospholipid synthesis. (**A**) The relative enrichments of each lipid class in shCtrl and PLPP1 knockdown A549/DDP cells with or without the Z-ligustilide and cisplatin combination treatment. ns, not significant, *** *p* < 0.001. (**B**) Protein expression of p-AKT in shCtrl and PLPP1 knockdown A549/DDP and H460/DDP cells with or without the Z-ligustilide and cisplatin combination treatment. (**C**,**D**) PI3K kinase activity assay and PIP_3_ level measurements in shCtrl and PLPP1 knockdown A549/DDP and H460/DDP cells with or without the Z-ligustilide and cisplatin combination treatment. ns, not significant, ** *p* < 0.01 and *** *p* < 0.001.

Phosphatidate phosphatase was reported to be able to regulate phospholipid synthesis [31,32]. LC-MS-based metabolomics showed that PLPP1 silence increased the content of metabolites during phospholipid synthesis in A549/DDP cells. Furthermore, Z-ligustilide+cisplatin increased the synthesis of phospholipids in PLPP1-knockdown A549/DDP cells compared to the control A549/DDP cells (Figure S4). The relative levels of total PC, PE, PI, PG, PS and TG are shown in Figure 6A. Various components of phospholipids are involved in the activation of AKT [33,34,35]. The Western blot experiment confirmed that Z-ligustilide+cisplatin decreased the level of phospho-AKT (p-AKT); however, PLPP1 silence reversed the expression of p-AKT in A549/DDP and H460/DDP cells (Figure 6B). Subsequently, we examined the phosphoinositide 3-kinase (PI3K) kinase activity and PIP_3_ levels based on the enzyme-linked immunosorbent assay (ELISA). The results showed that Z-ligustilide+cisplatin had little effect on the PI3K kinase activity in cisplatin-resistant lung cells and PLPP1 knockdown cisplatin-resistant lung cells (Figure 6C). However, Z-ligustilide+cisplatin decreased the levels of PIP_3_ in cisplatin-resistant lung cells, and the knockdown of PLPP1 increased the PIP_3_ levels in cisplatin-resistant lung cells treated with Z-ligustilide+cisplatin (Figure 6D). The data demonstrated that Z-ligustilide+cisplatin induced the inactivation of AKT by inhibiting PLPP1-mediated phospholipid synthesis.

## 3. Discussion

Lung cancer is a disease that seriously threatens human life and health [36,37]. The early stage of lung cancer is relatively insidious, and the course of lung cancer develops rapidly. Most lung cancer patients are in the middle and late stages of the disease when they are diagnosed, and therefore cannot undergo surgical resection due to the best treatment time being missed [38]. At present, the treatment of NSCLC mostly involves platinum drug-based combination chemotherapy. The most widely used chemotherapy drug is cisplatin, and cisplatin has the advantages of a wide anti-cancer range and strong effects in the treatment of lung cancer. In the treatment process, cisplatin shows a good therapeutic effect, but its long-term application causes tumor cells to develop resistance, resulting in a poor therapeutic effect [39]. Therefore, in order to improve the effect of clinical treatment for patients with lung cancer, it is very important to find new drugs against drug resistance.

Traditional Chinese medicine has the advantages of easy availability, high efficacy and low toxicity [40]. Moreover, traditional Chinese medicines have become the focus of the development of anti-tumor auxiliary drugs as a unique treatment method [41]. Z-Ligustilide, also known as 3-butenyl-4,5-dihydro-1 (3H)-isobenzofuranone, is the main active ingredient of the Chinese medicine Angelica volatile oil. Concerning Z-ligustilide in chemotherapeutic drug resistance, only one study by Qi et al. reported that Z-ligustilide (50 μM) and tamoxifen (5 μM) sensitized tamoxifen-resistant breast cancer cells by inhibiting autophagy and enhancing DNA damage [42]. This study demonstrated that the cell cycle was arrested in the S phase and that cell apoptosis was increased in cisplatin-resistant lung cancer cells with the treatment of Z-ligustilide (120 μM)+cisplatin (20 μM). The results suggest that the drug concentrations and molecular mechanisms of Z-ligustilide involved in reducing chemoresistance in different types of cancers are different. 

It has been reported that cisplatin could be deactivated upon dissolution in DMSO stock solutions, but that cisplatin activity is not influenced by 3% DMSO when cisplatin is in aqueous or growth medium [43]. In our study, cisplatin was dissolved in N, N-Dimethylformamide (DMF), and Z-ligustilide was dissolved in dimethyl sulfoxide (DMSO). Whether the DMSO that is mixed prior to the combined application has an influence on cisplatin activity is unknown. To examine the effect, A549 cells were treated with cisplatin and different concentrations of DMSO for 24 h, and were detected using an CCK-8 assay. The results demonstrated that the 0.05% concentration of DMSO (used in our study) had no effect on cisplatin activity. Moreover, the effect of DMSO at concentrations of 0.1%, 0.5% and 1% on cell viability was almost the same (Figure S5). The results revealed that DMSO introduced in the combination studies did not affect the activity of cisplatin. 

In addition, we found that 120 μM of Z-ligustilide could reduce cell viability by 30% in A549 cells; however, Jiang et al. showed a 50% reduction in cell viability after a 24 h exposure to 60 μM of Z-ligustilide in A549 cells [18]. The difference may have been from the solvents and nutritional environment of the cells. In our study, we used DMSO as the blank solvent, while Jiang et al. used ethanol as the blank solvent. To determine the effect of the nutritional environment of cells, we cultured A549 cells in medium containing different serum concentrations without or with 60 μM of Z-ligustilide treatment for 24 h, and then measured the cell viability. Figure S6 showed that insufficient nutrients increased the toxicity of Z-ligustilide to A549 cells.

Complex and diverse lipids, such as phospholipids, glycolipids, sphingolipids, and sterols, as well as storage lipids and various lipid derivatives, are the main components of membrane structures [44,45]. Recent studies found that phospholipid remodeling plays an important role in cancer progression. In cancer cells, altered phospholipid metabolism promotes tumor development and drug resistance [36]. Phospholipids are composed of glycerophospholipids and sphingomyelins. Glycerophospholipids include PC, PE, PS, PG, PI and PA. Changes in PC, PE, and PS can alter the properties of cell membranes and affect signal transduction and biological processes, thereby affecting tumor development and drug resistance [46,47,48]. It has been reported that the content of membrane phospholipids is different in A549 and A549/DDP cells treated with 30 μM of cisplatin. The level of phosphomonoesters decreased in A549 cells when they were treated with cisplatin; however, the level of phosphomonoesters increased in A549/DDP cells treated with cisplatin [49]. In our study, we found that the contents of PC, PE and PS increased in cisplatin-resistant lung cancer cells compared to lung cancer cells. However, the synthesis of phospholipids was reduced in cisplatin-resistant lung cancer cells treated with Z-ligustilide+cisplatin.

PLPP1 catalyzed the formation of diacylglycerol (DAG) from phosphatidic acid (PA), and participated in lipid metabolism and signal transduction. It has been reported that the loss of the phospholipid phosphatase Pah1 in yeast reduces DAG production, resulting in a reduction in the level and synthesis of TGs, and an increase in the level and synthesis of phospholipids [31,32]. The function of PLPP1 related to cancer has not been reported. We discovered that PLPP1 was upregulated in cisplatin-resistant lung cancer cells with the treatment of Z-ligustilide+cisplatin compared with cisplatin-resistant lung cancer cells. A low expression of PLPP1 was strongly associated with poor prognosis. PLPP1 silence alleviated the effects on cell viability, cell cycle, cell apoptosis and phospholipid synthesis mediated by Z-ligustilide+cisplatin. Altered phospholipids are closely related to the activation of the AKT signaling pathway. It was recognized that p53 reduced the levels of PIP_3_ through regulating the content of phospholipids, ultimately inhibiting AKT phosphorylation and activation [34]. Moreover, phospholipid scramblase 4 (PLSCR4) knockdown increases lipid accumulation, leading to an increase in PIP_3_ levels and the activation of AKT [50]. In our study, the inhibition of p-AKT expression and a decrease in PIP_3_ levels occurred in cisplatin-resistant lung cancer treated with Z-ligustilide+cisplatin; however, the knockdown of PLPP1 reversed the effects. The results demonstrated that the decrease in phospholipids mediated by PLPP1 reduced the levels of PIP_3_, ultimately inhibiting the activation of AKT. Interestingly, the expression of PLPP1 decreased in cisplatin-resistant lung cells compared with lung cancer cells, and the knockdown of PLPP1 increased the cell viability of cisplatin-resistant cells. The results indicate that PLPP1 enhances the sensitivity of cisplatin-resistant cells. Further study revealed that PLPP1 inhibited the synthesis of phospholipids, and decreased the levels of PIP_3_ and p-AKT. Altogether, these outcomes suggest that PLPP1 restores the sensitivity of cisplatin-resistant cells through the phospholipids–AKT axis. However, the effect of PLPP1 on cisplatin resistance in vivo, as well as the key factors or other compounds that regulate PLPP1 expression in cisplatin-resistant cells, remain unclear; further research including animal experiments around the above issues is necessary in the future.

In summary, the therapeutic effect of the combination of Z-ligustilide and cisplatin on cisplatin resistance in lung cancer and its action mechanism were thoroughly investigated via a cell biological experiment and metabolomics and transcriptomics techniques in the present study. Our findings demonstrated that Z-ligustilide obviously restored the inhibitory effect of cisplatin on cisplatin-resistant lung cancer. Z-ligustilide+cisplatin induced cell cycle arrest and apoptosis. Moreover, Z-ligustilide+cisplatin decreased phospholipid synthesis by upregulating the expression of PLPP1. Finally, the inhibition of the PIP3/AKT axis restored the cisplatin sensitivity of cisplatin-resistant lung cancer cells. This research provided a theoretical basis for the clinical treatment of lung cancer to restore the sensibility of chemotherapy, and also suggested a new idea for adjuvant treatment with traditional Chinese medicine.

## 4. Materials and Methods

### 4.1. Cell Culture

293T cells and A549 (human lung adenocarcinoma cell line) were purchased from Shanghai Cell Bank of Chinese Academy of Sciences (Shanghai, China), and A549/DDP (cisplatin-resistant lung adenocarcinoma cell line) was purchased from BeNa Biotechnology Co., Ltd. (Xinyang, China). H460 (human lung cancer cell line) and H460/DDP (cisplatin-resistant lung cancer cell line) were purchased from YaJi Biological (Shanghai, China). These cells were cultured in DMEM/high glucose medium or RPMI 1640, containing 10% fetal bovine serum, 100 units/mL penicillin and 100 μg/mL streptomycin in an incubator with 5% CO_2_ 37 °C.

### 4.2. Reagents and Antibodies

Cisplatin (Cat. No. HY-17394) and Z-ligustilide (Cat. No. HY-N0401A) were purchased from MedChemExpress Company (Shanghai, China). Cisplatin was dissolved in N,N-Dimethylformamide (DMF), and the stock solution concentration was 40 mM. Z-ligustilide was dissolved in dimethyl sulfoxide (DMSO), and the stock solution concentration was 240 mM. DMF at the concentration of 0.05% (*v*/*v*) and DMSO at the concentration 0.05% (*v*/*v*) were used as the control when the drug concentration was 0.

The following primary antibodies for BAX (Cat. No. 50599-2-Ig; Proteintech Technology, Wuhan, China), PLPP1 (PPAP2A, which was used in western blot experiments) (Cat. No. 17967-1-AP; Proteintech Technology, Wuhan, China) Cyclin A2 (Cat. No. 18202-1-AP; Proteintech Technology, Wuhan, China), AKT (Cat. No. 4691; Cell Signaling Technology, Boston, MA, USA), phosphor-Akt (Ser473) (Cat. No. 4060; Cell Signaling Technology, Boston, MA, USA), Cleaved Caspase-3 (Cat. No. 9661; Cell Signaling Technology, Boston, MA, USA), Caspase-3 (Cat. No. 9662; Cell Signaling Technology, Boston, MA, USA) GAPDH (Cat. No. 5174; Cell Signaling Technology, Boston, MA, USA), Cyclin E1 (Cat. No. ET1612-16; HUABIO Technology, Hangzhou, China) and PPAP2A (PLPP1, which was used in immunohistochemistry experiments) (Cat. No. NBP1-59011; Novus Biologicals, Littleton, CO, USA) were used.

### 4.3. Plasmid Construction and Transfection

DNA duplexes targeting PLPP1 short hairpin RNAs were cloned into the AgeI and EcoRI sites of the pLKO.1 puro vector. The target sequences for PLPP1 were shPLPP1#1 (5′-GAGGGAATGCAGAAAGAGTTA-3′) and shPLPP1#2 (5′-GTCTTGTTGCCGTATCCATTT-3′). For lentivirus packing, PLPP1 shRNAs and scramble shRNA plasmids were transfected into the 293T cell with packing plasmids (psPAX2 and pVSVG) using lipofectamine 2000 (Cat. No. 11668; Invitrogen, Carlsbad, CA, USA), according to the manufacturer’s instructions. Virus supernatant was collected at 48 h and 72 h after transfection, and was then infected into A549/DDP and H460/DDP cells for 24 h. Puromycin (1 μg/mL) was used to screen positive cells, and stable cells of PLPP1 silencing were established.

### 4.4. Cell Counting Kit-8 Assay

Cell viability was detected using a cell counting kit-8 (CCK-8) kit (Beyotime, Shanghai, China). Briefly, cells were seeded in 96-well plates containing 3 × 10^3^ cells and 100 μL of medium per well. After the cells adhered, different doses of Z-ligustilide and cisplatin were added to stimulate the cells for 24 h. Then, 10 μL of CCK-8 solution was added to each well, and placed in a 37 °C incubator for 1 h in the dark. Then, the absorbance was measured at 450 nm.

### 4.5. Cell Apoptosis Assay

A PE Annexin V Apoptosis Detection Kit (BD Pharmingen, San Diego, CA) was used to assess the percentage of apoptotic cells. Firstly, the cells were treated with cisplatin and Z-ligustilide alone or in combination for 24 h. Then, the cells were washed with PBS, digested with trypsin, and centrifuged. Next, 200 μL of 1X Binding Buffer was added to disperse the cells, and 5 μL of PE Annexin V and 5 μL of 7-AAD were added to the mix gently; then, the cells were incubated at room temperature for 15 min away from light. Finally, the apoptotic cell rates were analyzed using flow cytometry.

### 4.6. Cell Cycle Assay

Cisplatin- and Z-ligustilide-stimulated cells were fixed with prechilled 70% ethanol overnight, and then the cells were analyzed using a Cycle and Apoptosis Analysis Kit (Beyotime, Shanghai, China) according to the manufacturer’s instructions.

### 4.7. Liquid Chromatography-Mass Spectrometry Analysis 

The cells were seeded in 10 cm dishes and stimulated with cisplatin and Z-ligustilide alone or in combination for 24 h. The cells were washed three times with 10 mL of PBS, and quenched with liquid nitrogen. Next, cells were collected with 1 mL of extractant (80% methanol containing internal standard), added to chloroform and ultrapure water sequentially, and vortexed for 30 s. They were then centrifuged at 13,000× *g* for 15 min at 4 °C. Then, a 400 μL hydrophobic layer was aspirated and then lyophilized. Quality control (QC) samples were lyophilized in the same volume. First, 30 μL of chloroform/methanol (2:2, v:v) solution was added and vortexed for 30 s to reconstitution; then, 70 μL of acetonitrile/isopropanol/MilliQ water (65:30:5) containing 5 mM of ammonium acetate was added, and vortexed for 30 s to dilution. 

UPLC-Q-Exactive HF MS was used for nontargeted lipidomic analysis. The column was an ACQUITY UPLC C8 Column (100 × 2.1 mm, 1.7 μm). The column temperature was 60 °C and the flow rate was 0.3 mL/min. The injection chamber temperature was 10 °C. The injection volume was 5 μL. Mobile phase A was 60% acetonitrile/MilliQ water containing 10 mM of ammonium acetate, and phase B was 10% acetonitrile/isopropanol containing 10 mM of ammonium acetate. The mass spectrometry ion source sheath gas flow rate was 45 arb, and the auxiliary gas flow rate was 10 arb. The spray voltage was 3.5 kV (ESI+) and 3.0 kV (ESI-). The capillary temperature was 320 °C, and the auxiliary gas temperature was 350 °C. The resolution was 12 × 10^4^. The full scanning modes were used, and the scan ranges were 200–1100 *m*/*z* (ESI+) and 120–1600 *m*/*z* (ESI-).

### 4.8. RNA Sequencing

Total RNA was isolated using a TRIzol total RNA extraction kit (Tiangen, Beijing, China), which yielded > 2 μg of total RNA per sample. The RNA quality was examined via 0.8% agarose gel electrophoresis and spectrophotometry. Illumina HiSeq library construction was performed according to the manufacturer’s instructions (Illumina, San Diego, CA, USA). Oligo-dT primers were used to transverse mRNA to obtain cDNA (APExBIO, Houston, TX, USA). The second cDNA strand was synthesized by amplifying the cDNA, and was purified using magnetic beads. After library construction, library fragments were enriched via PCR amplification and selected according to a fragment size of 350–550 bp. The library was quality-assessed using an Agilent 2100 Bioanalyzer (Agilent, Santa Clara, CA, USA). The library was sequenced using the Illumina NovaSeq 6000 sequencing platform (Paired end150) to generate raw reads.

### 4.9. Western Blot

Cells were harvested and lysed with RIPA (Beyotime, Shanghai, China) containing protein and phosphatase inhibitors (APExBIO, Houston, TX, USA). The total protein extract (20 μg) was loaded in 4–20% SDS-PAGE. The protein was transferred to a PVDF membrane, and was blocked with 5% milk. Subsequently, the protein was incubated with primary antibodies and second HRP-linked antibodies. Finally, the protein was visualized using ECL Chemiluminescence Chromogenic Solution (Tanon, Shanghai, China).

### 4.10. Real-Time PCR

Total RNA was isolated with the Trizol Reagent Kit (Takara, Tokyo, Japan) according to the manufacturer’s instructions. The cDNA was generated by using the PrimeScript RT reagent Kit with gDNA Eraser (Takara, Tokyo, Japan). Real-time PCR was carried out by using TB Green Premix Ex Taq II (Takara, Tokyo, Japan) operating on the LightCycler 96 Real-time PCR system. The primers sequencings were as follows:

GAPDH (forward): TCCAAAATCAAGTGGGGCGA

GAPDH (reverse): TGATGACCCTTTTGGCTCCC

Cyclin A2 (forward): GATTTCGTCTTCCAGCAGCAG

Cyclin A2 (reverse): ATCTGACAAGCATCGGGACC

Cyclin E1 (forward): AGAGGAAGGCAAACGTGACC

Cyclin E1 (reverse): GAGGCTTGCACGTTGAGTTT

BCL2 (forward): GAACTGGGGGAGGATTGTGG

BCL2 (reverse): CCGTACAGTTCCACAAAGGC

BAX (forward): GACATTGGACTTCCTCCGGG

BAX (reverse): GGGACATCAGTCGCTTCAGT

PLPP1 (forward): CTGGCTGGATTGCCTTTTGC

PLPP1 (reverse): AACAGACAGGGTTTCTCCAAGA

### 4.11. Immunohistochemistry

The human lung cancer tissue chip was purchased from Outdo Biotech (Shanghai, China). The tissue chip used was HLugA180Su04.

Immunostaining was performed by using the Universal SP Kit (SP-9000; ZSGB-BIO, Beijing, China) following the instructions. The primary antibody for PLPP1 (known as PPAP2A, Cat. No. NBP1-59011; Novus Biologicals, Colorado, USA) was used for immunohistochemical staining, and the dilution ratio of the PLPP1 antibody was 1:200. The staining intensity was divided into 4 categories: no signal (0), weak (1), moderate (2), and marked (3). The percentage score was divided into 4 categories: 1–25% (1), 26–50% (2), 51–75% (3), and 76–100% (4). The staining score was calculated by multiplying the staining intensity and percentage score. A score of 0 was regarded as negative expression (−), ≤ 4 was regarded as lower expression (+), 5–8 was regarded as moderate expression (++), and ≥ 9 was regarded as high expression (+++). Based on the optimal cutoff value, a score ≤ 4 was used to define tumors of low expression, and score ≥ 5 for high expression.

### 4.12. PIP_3_ Measurements

PIP_3_ levels were analyzed using a Human PIP_3_ ELISA Kit according to the manufacturer’s instructions (COIBO BIO, Shanghai, China). 

### 4.13. PI3K Kinase Assay

PI3K kinase activity was examined using a Human PI3K ELISA Kit according to the manufacturer’s instructions (COIBO BIO, Shanghai, China). 

### 4.14. Statistical Analysis

The experiments were repeated three or more times. Data are presented as mean ± SD. Statistical significance was measured via Student’s t-test (unpaired, two-tailed), with *p* < 0.05 indicating significance. Statistical analysis was performed with Prism 6 software (La Jolla, CA, USA), FlowJo 10.6.2 software (TreeStar, Ashland, OR, USA), SIMCA-P 14.1 software (Umetrics, Umea, Sweden) and the Multi Experiment Viewer (http://www.tm4.org) (accessed on 25 December 2021).

## Data Availability

Data will be made available upon request.

## Supplementary Materials

The following supporting information can be downloaded at: https://www.mdpi.com/article/10.3390/ijms242317046/s1.
