# Supplementary material for: Z-Ligustilide Combined with Cisplatin Reduces PLPP1-Mediated Phospholipid Synthesis to Impair Cisplatin Resistance in Lung Cancer
Source: Int J Mol Sci. 2023 Dec 1;24(23):17046. doi: 10.3390/ijms242317046 (PMC10706864; doi:10.3390/ijms242317046)
Supplement: Supplementary file 1 [file ijms-24-17046-s001.zip › ijms-2544119-supplementary.pdf]

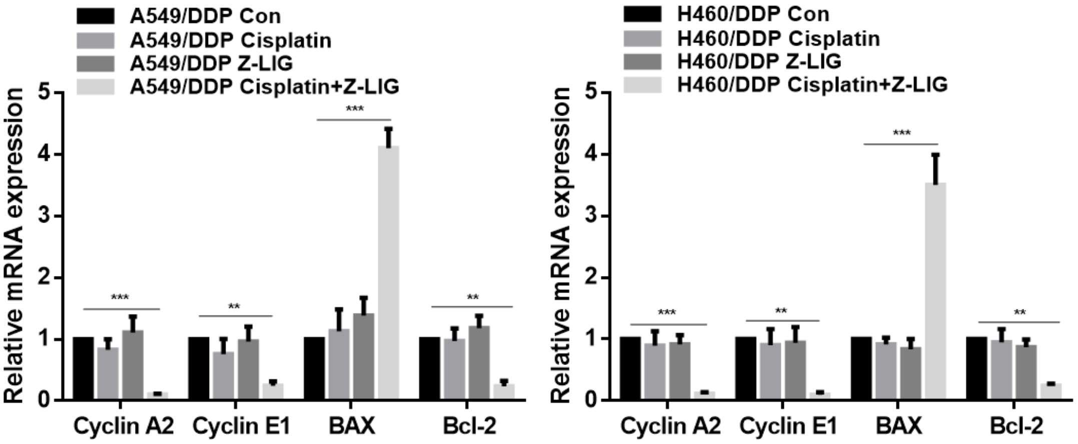

Figure S1. The Z-ligustilide+cisplatin induced cell apoptosis. mRNA expression of Cyclin A2, Cyclin E1, BAX and Bcl-2 in A549/DDP and H460/DDP, A549/DDP and H460/DDP treated with cisplatin and Z-ligustilide alone or in combination. $**p < 0.01$ and $***p < 0.001$.

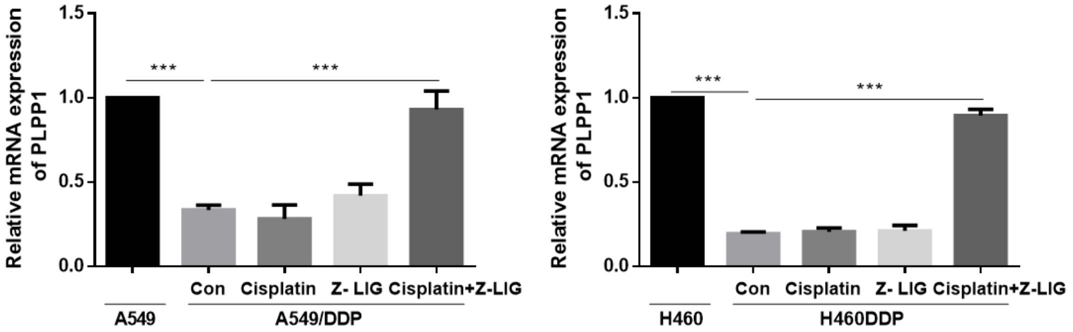

Figure S2. The Z-ligustilide+cisplatin reversed the expression of PLPP1. mRNA level of PLPP1 was determined by real-time PCR in A549 and H460, A549/DDP and H460/DDP, A549/DDP and H460/DDP treated with cisplatin and Z-ligustilide alone or in combination. ***$p < 0.001$.

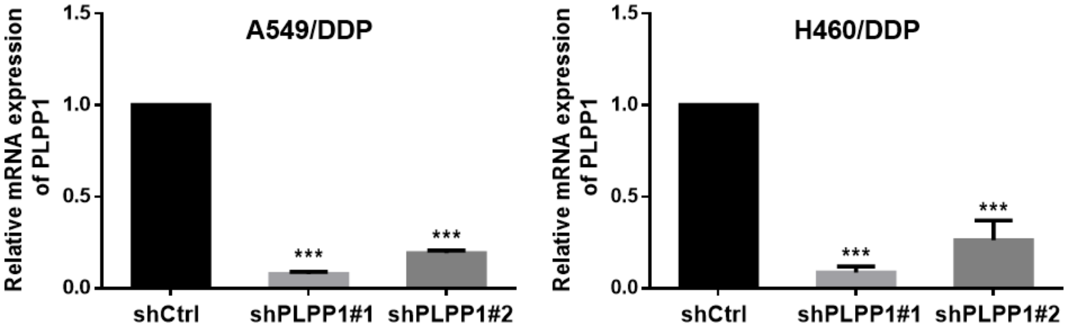

Figure S3. The validation of PLPP1 knockdown efficiency. mRNA expression of PLPP1 in shCtrl and PLPP1 knockdown A549/DDP and H460/DDP cells, respectively. ***$p < 0.001$.

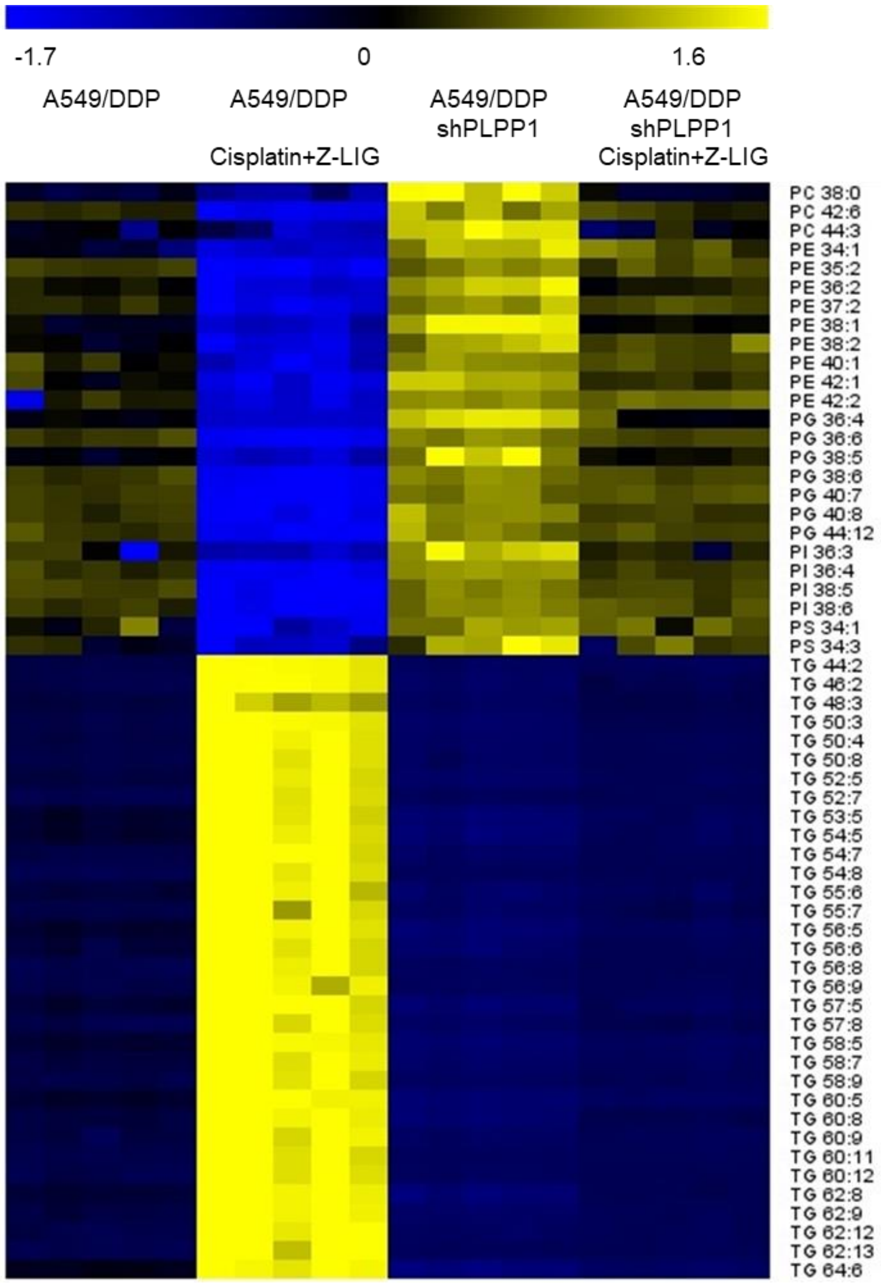

Figure S4. Heat map of the differential lipids in shCtrl and PLPP1 knockdown A549/DDP cells with or without Z-ligustilide+cisplatin treatment.

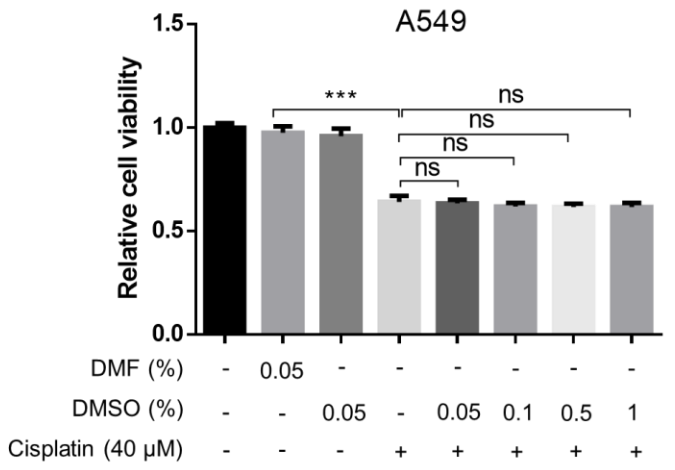

Figure S5. Cell viability assay of A549 cells. 40 μM cisplatin and different concentrations (i.e., 0.1%, 0.5% and 1%) of DMSO were respectively added to A549 cells for 24 hours, and cell viability was detected by CCK-8 assay. ns, no significant, ***$p < 0.001$.

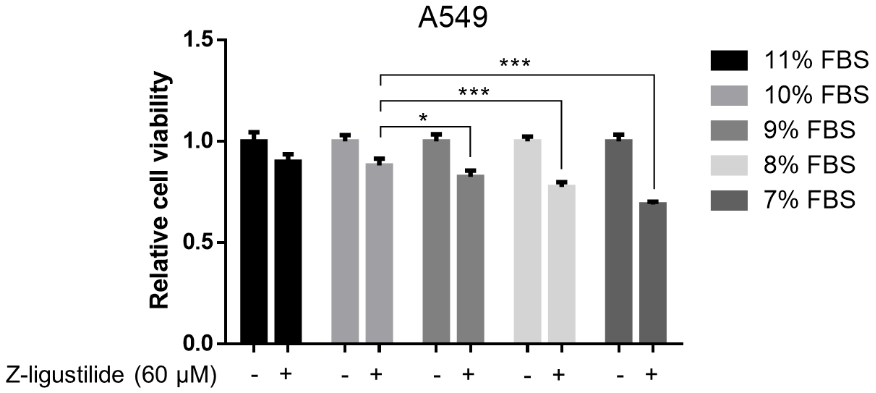

Figure S6. Cell viability assay of A549 cells. A549 cells were treated with different concentrations of serum without or with Z-ligustilide for 24 h, cell viability was examined by CCK-8 assay. *$p < 0.05$, ***$p < 0.001$.
